# Co-Enrichment of *Desulfovibrio* and *Erysipelotrichaceae* and Dysregulation of Multiple Amino Acid Pathways in HFpEF: Insights into TXG-Associated Metabolic Changes

**DOI:** 10.3390/ijms27198445

**Published:** 2026-09-22

**Authors:** Wei Zhang, Yiyi Zhu, Xiaosu Chu, Xinyi Zhang, Zhuo Pan, Aiying Li

**Affiliations:** 1 Department of Biochemistry and Molecular Biology, College of Pharmacy, Hebei University of Chinese Medicine, Shijiazhuang 050200, China; 2 Hebei Key Laboratory of Chinese Medicine Research on Cardio-Cerebrovascular Disease, Shijiazhuang 050091, China

**Keywords:** gut microbiota, *Erysipelotrichaceae*, *Desulfovibrio*, metagenomics, branched-chain amino acid biosynthesis, total xanthones of *Gentianella acuta*, heart failure with preserved ejection fraction

## Abstract

Gut enrichment of *Desulfovibrio* and *Erysipelotrichaceae* may be associated with systemic amino acid metabolic dysfunction, myocardial injury, and heart failure with preserved ejection fraction (HFpEF). These taxa appear to influence host amino acid metabolism through distinct processes, but the functional implications of their co-enrichment in HFpEF remain unclear. Total xanthones from *Gentianella acuta* (TXG) have documented cardioprotective effects, although their effects on gut microbiota and systemic metabolism remain unexplored. We established a mouse model of HFpEF using a high-fat diet (HFD) combined with Nω-nitro-L-arginine methyl ester (L-NAME). We integrated 16S rRNA sequencing, fecal metagenomics, and targeted serum amino acid metabolomics to examine microbe–metabolite associations, with echocardiography and histopathology providing complementary phenotypic evidence. Microbial and metabolomic analyses revealed pronounced enrichment of *Desulfovibrio* and *Erysipelotrichaceae* (particularly *Dubosiella*), accompanied by elevated serum isoleucine, tryptophan, and homocysteine levels. TXG treatment was associated with lower abundance of both taxa, altered serum amino acid profiles, preserved colonic barrier integrity, and reduced myocardial inflammation and fibrosis. These changes coincided with improved diastolic function. Together, these findings associate co-enrichment of the two taxa with dysregulation of multiple amino acid pathways in HFpEF. TXG treatment was accompanied by attenuated cardiac and metabolic injury as well as shifts in gut-microbiota profiles, with these correlative observations motivating further investigation of *Gentianella acuta* for metabolic heart failure.

## 1. Introduction

Gut taxa, including *Desulfovibrio* and *Erysipelotrichaceae*, are associated with systemic metabolic dysfunction, altered amino acid metabolic pathways, and cardiac diastolic dysfunction in heart failure with preserved ejection fraction (HFpEF) mouse models. Research on the gut–heart axis has implicated microbial imbalance as a potential contributor to cardiometabolic injury. However, the distinct functions and concurrent alterations of these two bacterial groups have received less attention [1,2]. HFpEF is a heterogeneous metabolic cardiovascular disease for which specific therapeutic options remain limited [3,4,5].

Gut microbiota dysbiosis has been proposed as an early feature of gut-derived injury in HFpEF [6,7,8]. *Erysipelotrichaceae* and *Desulfovibrio* are two functionally distinct bacterial groups with different metabolic characteristics. *Erysipelotrichaceae* can metabolize amino acid derivatives through anaerobic respiration [9]. More broadly, alterations in the gut microbiome are consistently associated with metabolic diseases [10,11,12,13]. *Desulfovibrio* is a sulfate-reducing bacterial genus associated with endotoxin and H_2_S production. H_2_S has been implicated in impaired host metabolism [14], whereas endotoxin has been linked to intestinal barrier dysfunction and systemic low-grade inflammation [15]. These bacterial groups may therefore link gut microbial dysregulation to cardiac injury.

Most research on the gut–heart axis has focused on heart failure with reduced ejection fraction (HFrEF) [16]. In the metabolic HFpEF model induced by a high-fat diet (HFD) and Nω-nitro-L-arginine methyl ester (L-NAME), metabolic disturbances associated with *Erysipelotrichaceae* and *Desulfovibrio* have not been systematically characterized using multi-omics. Total xanthones extracted from *Gentianella acuta* (TXG) have documented cardioprotective activities [17,18,19,20]. However, their effects on these taxa and the associated disturbances in amino acid metabolism remain uncharacterized [21].

Given their distinct biological characteristics, we hypothesized that co-enrichment of *Desulfovibrio* and *Erysipelotrichaceae* may be associated with three microbial amino acid synthesis modules. These associations could contribute to systemic metabolic disturbances and HFpEF phenotypes, while TXG may alter microbial community structure and metabolic profiles. We examined three aspects of metabolic dysregulation. The first was altered tryptophan metabolism, reflected by elevated circulating tryptophan levels and increased abundance of microbial biosynthesis genes. Whether and how these changes contribute to host pathophysiology remains unclear [22,23,24]. The second was enrichment of bacterial branched-chain amino acid (BCAA) biosynthetic pathways and their association with serum isoleucine. Disturbances in BCAA metabolism have been linked to cardiac dysfunction [25,26,27]. The third was altered sulfur-containing amino acid metabolism and its association with homocysteine accumulation. Homocysteine has been linked to endothelial injury and cardiovascular disease [28,29]. Previous studies suggest that these metabolic alterations are associated with the gut–heart axis and myocardial injury [30,31,32].

Given the above knowledge gaps, the present study aimed to characterize gut enrichment of *Erysipelotrichaceae* and *Desulfovibrio* in a mouse HFpEF model using multi-omics approaches. We further sought to investigate their correlations with disturbances in BCAA, tryptophan and sulfur-containing amino acid metabolism, and to evaluate whether TXG could restore gut microbial homeostasis, ameliorate amino-acid metabolic dysregulation and improve cardiac phenotypes.

## 2. Results

### 2.1. TXG Fingerprint and Experimental Design

We first characterized the chemical composition of TXG. The HPLC fingerprint showed multiple characteristic peaks within a defined retention time range (Figure 1A). These peaks corresponded to those in a mixed standard containing five major xanthones: mangiferin, norswertianolin, swertianolin, demethylbellidifolin, and bellidifolin (Figure 1B). This correspondence confirmed the principal constituents of the extract. The Kekulé structures showed a shared dibenzo-γ-pyrone (xanthone) scaffold, with differences in the type, number, and position of substituents (Figure 1C). The MS/MS spectrum of mangiferin, a representative constituent, showed characteristic neutral losses and aglycone fragment ions, further supporting its structural identification (Figure 1D). We then established an HFpEF mouse model using a HFD combined with L-NAME. Mice were assigned to four groups: CON, MOD, TRE (4 mg/kg TXG), and HIG (40 mg/kg TXG). Outcomes were assessed after 6 weeks of intervention. Figure 1E summarizes the experimental protocol.

### 2.2. TXG Attenuates Myocardial Injury in the HFpEF Model

We assessed cardiac histology and molecular markers to evaluate myocardial injury in the HFpEF model. Basic physiological data (body weight, blood pressure and blood glucose) and cardiac Doppler echocardiography results are shown in Figure S1 and Figure S2, respectively. Gross examination showed cardiac hypertrophy in the MOD group, which was less pronounced following TXG treatment (Figure 2A). Western blotting showed significantly increased myocardial levels of the pro-inflammatory protein IL-18 in MOD mice. TXG treatment suppressed this increase (Figure 2B,C).

Hematoxylin and Eosin (H&E) staining showed marked disruption of myocardial architecture, cardiomyocyte disorganization, and inflammatory infiltration in the MOD group. These changes were attenuated by TXG treatment (Figure 2D). Masson’s trichrome staining revealed extensive perivascular collagen accumulation and interstitial fibrosis in model mice; TXG markedly reduced perivascular collagen deposition (Figure 2E). Picrosirius red staining also showed abnormal perivascular collagen deposition and an elevated type I/III collagen ratio in the MOD group. TXG treatment reversed these changes (Figure 2F). Collagen fraction from histological slices is presented in Figure S3A,B. Together, these findings indicate that TXG attenuated myocardial inflammatory infiltration and interstitial fibrosis in HFpEF mice.

### 2.3. TXG Is Associated with Altered Gut Microbiota Structure and Reduced Abundance of Key Disease-Associated Taxa in the HFpEF Model

Fecal samples underwent 16S rRNA sequencing to characterize gut microbiota dysbiosis in HFpEF and changes associated with TXG treatment. Four groups were included in the in vivo phenotypic and safety assessments, but only CON, MOD, and TRE were selected for amplicon sequencing. The microbiota analysis focused on differences in community composition among these groups. The high-dose group was included in physiological, biochemical, and pathological safety assessments but not in microbiota sequencing.

Gut microbiota diversity and community structure. Alpha diversity did not differ significantly among the CON, MOD, and TRE groups (Figure 3A). Principal coordinate analysis (PCoA) showed separation between the CON and MOD microbial communities, with TRE samples shifted toward CON samples (Figure 3B). Global permutational multivariate analysis of variance (PERMANOVA) indicated significant group differences (*R*^2^ = 0.4814, *p* = 0.001), whereas PERMDISP provided no evidence of differences in dispersion (*p* = 0.603). The ternary plot showed the distribution of *Bacillota*, *Bacteroidota*, and *Pseudomonadota* relative abundance across the three groups (Figure 3C). The MOD group had a higher proportion of *Pseudomonadota* and a lower proportion of *Bacteroidota*. The community composition river plot further illustrated between-group differences at the study endpoint (Figure 3D).

Gut microbiota correlation networks and key disease-associated taxa. Genus-level correlation networks showed greater complexity and connection density in MOD mice than in CON mice. These changes were accompanied by an altered balance between positive and negative correlations (Figure 4A,B). Network topology also differed between the TRE and MOD groups. Linear discriminant analysis Effect Size (LEfSe) analysis identified differentially abundant taxa using an LDA score > 4.0, and the cladogram showed their phylogenetic distribution across taxonomic levels (Figure 4C). *Desulfovibrio* was the most significantly enriched Gram-negative sulfate-reducing taxon in the MOD group (Figure 4D). Its relative abundance was significantly higher in MOD mice than in CON mice and was lower in TRE mice following TXG treatment. *Erysipelotrichaceae*, particularly Dubosiella, was the most prominently enriched Gram-positive group in MOD mice (Figure 4E). Its relative abundance was significantly higher than in CON mice and was lower following TXG treatment. These findings identify *Erysipelotrichaceae* and *Desulfovibrio* as two functionally distinct taxa associated with the HFpEF phenotype, both of which were less abundant with TXG treatment.

### 2.4. TXG Is Associated with Altered Serum Amino Acid Profiles in the HFpEF Model

Based on the metagenomic evidence of altered microbial amino acid metabolism, we used targeted Ultra-Performance Liquid Chromatography–Tandem Mass Spectrometry (UPLC-MS/MS) to quantify serum amino acid profiles.

Overall metabolic profiles. Total ion chromatograms (TICs) showed well-separated peaks, stable signals, and reproducible retention times across samples (Figure 5A). Principal component analysis (PCA) showed a trend toward separation among the CON, MOD, and TRE serum metabolic profiles (Figure 5B). The first two principal components explained a large proportion of the total variance. These patterns suggest that HFpEF was associated with a distinct amino acid profile, with the TRE profile shifted toward that of CON.

Changes in key amino acids. The heatmap showed clustering of differentially abundant amino acids across the three groups (Figure 5C). MOD samples clustered separately from CON samples, while TRE samples occupied an intermediate position. Isoleucine showed the most significant change among the BCAAs. Serum isoleucine levels were significantly higher in MOD mice than in CON mice and lower in TRE mice than in MOD mice (Figure 5F). Leucine and valine showed similar upward trends, but these differences were not statistically significant. The associated metabolite–pathway relationships are shown in the Kyoto Encyclopedia of Genes and Genomes (KEGG) chord diagram (Figure 5D). For sulfur-containing amino acid metabolism, homocysteine levels were significantly higher in MOD mice than in CON mice and lower in TRE mice (Figure 5H). The corresponding pathway associations are shown in Figure 5E.

### 2.5. TXG Is Associated with Altered Abundance of Gut Microbial Amino Acid Metabolic Modules in the HFpEF Model

We performed fecal metagenomic analysis to characterize functional metabolic changes accompanying gut microbiota dysbiosis.

Differentially abundant genes and metabolic pathway enrichment. The volcano plot showed differences in amino acid metabolism and associated one-carbon methyl metabolism between the MOD and TRE groups (Figure 6A). Several amino acid metabolism-related genes, including iso_dh, 2-oxoacid_dh, and IGPS, were significantly less abundant in MOD mice. In contrast, features related to tryptophan biosynthesis (Trp_syntA), homoserine dehydrogenase (Homoserine_dh), MTHFR, and S-methyl transfer were more abundant in MOD mice. These features are involved in amino acid and one-carbon metabolism. The Venn diagram showed 831 shared differential molecular features, with 101 unique to MOD and 410 unique to TRE (Figure 6B). The GO Sankey diagram showed differences in the abundance of amino acid catalytic and membrane transport functions (Figure 6C). Catalytic molecular functions were more enriched in TRE mice, whereas amino acid biosynthetic biological processes were overrepresented in MOD mice. The KEGG hierarchical Sankey plot identified amino acid metabolism as the most divergent Level 2 pathway within the dominant Level 1 category, Metabolism (Figure 6D). Gene sets related to overall metabolic pathways were predominantly enriched in TRE mice. MOD mice showed greater relative abundance of genetic information processing and cellular processes closely linked to amino acid synthesis. Together, these annotations suggest that altered amino acid and associated one-carbon methyl metabolism may distinguish the MOD and TRE groups.

### 2.6. TXG Is Associated with Reduced Intestinal Barrier Injury in the HFpEF Model

We evaluated colonic histopathology to determine whether microbial and metabolic disturbances were accompanied by pathological changes beyond the heart.

Colonic structure and intestinal barrier function. H&E staining showed intact colonic mucosa, orderly epithelial cells, and no evident inflammatory infiltration in the lamina propria of CON mice. MOD mice showed disrupted mucosal architecture, epithelial injury and sloughing, diffuse inflammatory infiltration in the lamina propria, and marked mucosal thinning. These changes were less pronounced in TXG-treated mice. Inflammation scores were significantly higher in MOD mice than in CON mice and significantly lower in both TRE and HIG mice than in MOD mice (Figure 7D). Periodic Acid-Schiff and Alcian Blue (PAS-AB) staining showed abundant goblet cells and mucus in CON mice, with a continuous protective layer of positive staining. MOD mice had fewer goblet cells and significantly reduced mucus secretion. Goblet cell numbers and mucus secretion were higher in TXG-treated mice. The percentage of PAS-AB-positive area was significantly lower in MOD mice than in CON mice and significantly higher in TXG-treated mice than in MOD mice (Figure 7E and Figure S3C).

## 3. Discussion

We identified pronounced co-enrichment of *Erysipelotrichaceae* and *Desulfovibrio* in HFpEF mice, associated with disturbances in multiple amino acid pathways and myocardial injury. Mice receiving HFD and L-NAME exhibited diastolic dysfunction, myocardial inflammation, and interstitial fibrosis, together with gut microbiota dysbiosis and intestinal barrier damage. *Erysipelotrichaceae* and *Desulfovibrio* were prominent disease-associated taxa enriched in these mice. Their enrichment was associated with altered tryptophan metabolism, elevated BCAAs such as isoleucine, and homocysteine accumulation. These changes may link gut microbial disturbances to myocardial injury. TXG treatment was accompanied by lower abundance of both taxa, improved intestinal barrier integrity, and altered amino acid profiles. These changes coincided with attenuated myocardial injury and improved left ventricular diastolic function [33,34].

Gut microbiota dysbiosis can involve enrichment of specific functional groups whose distinct metabolic characteristics may contribute to disease progression. *Erysipelotrichaceae* is a Gram-positive family within the phylum *Bacillota* [35]. Its cell wall lacks lipopolysaccharide (LPS), and its disease-associated effects are linked to host metabolic homeostasis rather than endotoxin production. Previous studies have associated HFD-induced *Erysipelotrichaceae* enrichment with obesity and insulin resistance through altered intestinal bile acid metabolism and increased energy extraction. This family is therefore a characteristic taxon in metabolic syndrome-associated dysbiosis [9,10,11,27,36]. In our study, *Erysipelotrichaceae* enrichment coincided with increased abundance of BCAA biosynthesis pathways in the metagenome and higher circulating amino acid levels. This association suggests that its abundance correlates with intestinal BCAA-related gene abundance in the gut microbiome. *Erysipelotrichaceae* overgrowth has also been associated with altered mucosal microbial communities and local innate immune activation by peptidoglycan and lipoteichoic acid. The resulting low-grade mucosal inflammation may affect barrier integrity and facilitate the entry of metabolic factors and inflammatory mediators into the circulation. *Desulfovibrio*, a Gram-negative sulfate-reducing bacterial genus, has instead been linked to barrier disruption and inflammatory activation [14,15]. Its cell wall components have been associated with systemic low-grade inflammation and may contribute to inflammatory signaling along the gut–heart axis. Much of the mechanistic reasoning described in the following paragraph is derived from the previously published literature; these mediators were not directly quantified in the present study. According to previous studies, excessive H_2_S production has been linked to disrupted epithelial tight junctions, increased mucosal permeability, and translocation of bacterial products. These effects could sustain a cycle of barrier injury and inflammatory activation [14,15]. In our dataset, *Desulfovibrio* enrichment was associated with altered intestinal sulfur-containing amino acid metabolism. Sulfur transformations mediated by this genus have been linked to accumulation of metabolites such as cysteine and homocysteine, which other studies associate with cardiovascular changes. Thus, *Erysipelotrichaceae* was primarily associated with metabolic disturbances, whereas *Desulfovibrio* was primarily associated with barrier-related changes. These parallel metabolic and structural alterations may contribute to the observed dysregulation of the gut–heart axis.

Co-enrichment of *Erysipelotrichaceae* and *Desulfovibrio* was associated with disturbances across multiple amino acid pathways, which may correlate with systemic cardiovascular phenotypes observed in this HFpEF model. Microbial tryptophan metabolism reflects the functional state of the gut microbial community. Under physiological conditions, two functionally distinct pathways coexist to maintain metabolic balance. In the HFpEF model, elevated serum tryptophan and increased abundance of microbial tryptophan biosynthesis genes accompanied altered microbiota structure. These findings suggest altered tryptophan metabolism but do not directly establish changes in metabolic flux. Gut microbiota-derived tryptophan metabolites have been reported to include both protective and potentially harmful compounds [16,17,18,37,38,39]. However, these downstream metabolites were not measured here; therefore, no inference about pathway shifts or their contribution to myocardial injury can be made. Targeted measurements are needed to test this hypothesis and its possible association with myocardial injury.

Metagenomic analysis showed significantly greater abundance of microbial BCAA biosynthesis pathways in MOD mice, accompanied by significantly elevated serum isoleucine. Isoleucine was the most consistently and significantly altered of the three BCAAs, suggesting that it may be a representative marker of BCAA metabolic disturbance in this model. Previous studies have associated sustained isoleucine accumulation with myocardial mTOR signaling, cardiomyocyte hypertrophy, fibroblast activation, and collagen deposition [19,20,21,22]. Elevated isoleucine has also been linked to altered myocardial energy metabolism and worsened myocardial insulin resistance, potentially interacting with HFD-associated systemic metabolic changes. *Erysipelotrichaceae* enrichment coincided with greater abundance of microbial BCAA biosynthesis pathways and elevated serum isoleucine. These parallel changes suggest an association among *Erysipelotrichaceae* abundance, microbial BCAA-related gene abundance, and circulating isoleucine levels.

These three metabolic pathways reflect different functional aspects of altered microbial community structure. Their association with inflammatory and fibrotic signaling suggests an interconnected pathological network. This network may partly explain multisystem involvement in HFpEF and the limited efficacy of therapies directed at a single target [4,37].

The effects of TXG in HFpEF may involve associations across gut microbiota, amino acid metabolism, and cardiac outcomes. TXG treatment was associated with lower relative abundance of *Erysipelotrichaceae* and *Desulfovibrio*, improved colonic mucosal structure, altered mucus composition, and greater intestinal barrier integrity. These changes may influence signaling along the gut–heart axis. The concurrent changes in Gram-positive and Gram-negative disease-associated taxa are a notable feature of TXG-associated microbial alterations [29,30,31,40]. At the metabolic level, TXG treatment was associated with reduced BCAA biosynthesis pathway abundance, lower serum isoleucine and homocysteine levels, and altered tryptophan metabolism. Associations across several pathways may reflect the multiple constituents of TXG [29,30,31]. These microbial and metabolic changes were accompanied by lower levels of inflammatory mediators and metabolites, reduced myocardial inflammatory infiltration and fibrosis, and improved left ventricular diastolic function.

This study has several limitations. First, it used a mouse model, and microbial community structure and metabolic characteristics differ between mice and humans [38]. The roles of *Erysipelotrichaceae* and *Desulfovibrio* and the associated metabolic disturbances require validation in clinical HFpEF cohorts. Second, the observed relationships among microbiota, metabolites, and cardiac function are correlative. Future functional validation, including anaerobic culture of *Dubosiella* strains and assessment of BCAA-synthesizing capacity in vitro, will be required to further test this hypothesis.. Fecal microbiota transplantation, colonization with specific disease-associated bacteria, and metabolite supplementation could help establish causal roles and distinguish the contributions of individual amino acid pathways. Third, the specific microbial targets and active constituents of TXG remain unclear. Future studies could combine drug exposure profiling with validation of individual constituents to identify direct microbial targets and molecular mechanisms. Overall, the observed associations among bacterial co-enrichment, altered tryptophan metabolism, isoleucine accumulation, and elevated homocysteine provide preliminary evidence linking these taxa to metabolic and cardiac changes. TXG-associated changes in these taxa and metabolic profiles support further investigation of *G. acuta* in metabolic heart failure. Finally, metagenomic and metabolomic analyses included only three biological replicates per group. This small sample size limits statistical power and may leave the findings sensitive to interindividual variation. These results should therefore be interpreted as preliminary associations requiring validation in larger cohorts.

## 4. Materials and Methods

### 4.1. Materials

#### 4.1.1. In Vivo Animal Model

Male specific pathogen-free (SPF) C57BL/6J mice, aged 8 weeks and weighing 18–20 g, were purchased from Skebaisi Biotechnology Co., Ltd. (Zhengzhou, China; license number: SCXK (Yu) 2025-0005). Mice were housed in an SPF facility at 22 ± 2 °C and 50 ± 5% humidity under a 12 h light/dark cycle. They had free access to standard chow and water. Experiments began after 1 week of acclimatization. All procedures followed the guidelines for ethical review of laboratory animals and were approved by the Laboratory Animal Ethics Committee of Hebei University of Chinese Medicine (KJLL(D)20261034). All animal procedures complied with the ARRIVE 2.0 guidelines.

#### 4.1.2. Reagents and Instruments

The HFD (D12492, 60% kcal from fat) was purchased from Research Diets (New Brunswick, NJ, USA). Nω-nitro-L-arginine methyl ester (L-NAME, purity ≥ 98%) was obtained from Sigma-Aldrich (St. Louis, MO, USA). *G. acuta* was purchased from a medicinal materials market in Inner Mongolia and taxonomically identified by Hebei University of Chinese Medicine. The total xanthone extract was prepared by methanol reflux extraction, and HPLC confirmed a total xanthone purity exceeding 85%. H&E, Masson’s trichrome, and PAS-AB staining kits were purchased from Maishupu Biotechnology (Wuhan, China). The genomic DNA extraction kit (DP302) was obtained from Biomarker Technologies (Beijing, China). Reagents for targeted amino acid metabolomics were supplied by Yuanhe Biotechnology (Shanghai, China).

The main instruments were the Vevo 2100 small animal ultrasound imaging system (Visual Sonics, Toronto, ON, Canada); BP-2000 non-invasive blood pressure analysis system (Taimeng, Chengdu, China); OneTouch Ultra glucometer (Johnson & Johnson, New Brunswick, NJ, USA); XR-XB100 small animal treadmill (Xinruan, Shanghai, China); RM2265 paraffin microtome (Leica, Wetzlar, Germany); Illumina NovaSeq 6000 high-throughput sequencing platform (Illumina, San Diego, CA, USA); and a UPLC-MS/MS system (Shimadzu, Kyoto, Japan).

### 4.2. Methods

#### 4.2.1. Group Allocation and HFpEF Model Establishment

After acclimatization, mice were randomly assigned to four groups (*n* = 6 per group). The control group (CON) received normal chow and distilled water for 6 weeks. The model group (MOD) received HFD and distilled water containing 0.5 g/L L-NAME for 6 weeks. The TXG treatment group (TRE) received HFD and 0.5 g/L L-NAME in distilled water, together with daily oral TXG gavage (4 mg/kg/day) for 6 weeks. The high-dose group (HIG) received the same diet and L-NAME regimen, with daily oral TXG gavage at 40 mg/kg/day for 6 weeks. The gavage volume was 1 mL/kg in all groups; CON and MOD mice received an equal volume of normal saline.

HFpEF model criteria were assessed after the 6-week intervention. Echocardiographic criteria included ejection fraction (EF) ≥ 50%, no significant enlargement of left ventricular end-diastolic diameter (LVEDD) or end-systolic diameter (LVESD), and E/A ratio ≤ 1. A reduction in early diastolic mitral annular velocity (e’) with E/e’ ratio ≥ 15 was also required. Additional criteria were systolic blood pressure ≥ 140 mmHg, fasting blood glucose ≥ 6.1 mmol/L, and exercise capacity ≥ 30% lower than in CON mice. The model was considered established when all criteria were met [41].

#### 4.2.2. Physiological Monitoring

Body weight: Mice were weighed weekly using an electronic balance (accuracy: 0.01 g).

Blood pressure: Systolic blood pressure (SBP) and diastolic blood pressure (DBP) were measured weekly using a non-invasive tail-cuff system. Before measurement, mice were acclimatized in a 37 °C incubator for 10 min. Five consecutive measurements were obtained, and their mean was recorded.

Blood glucose: After the 6-week intervention, mice were fasted for 12 h. Fasting blood glucose (FBG) was measured in tail-tip blood using a glucometer.

Exercise capacity: After the 6-week intervention, mice underwent an exhaustive exercise test on a small animal treadmill. The initial speed was 5 m/min and increased by 1 m/min every 3 min. The test ended when a mouse contacted the electric shock grid three consecutive times and could no longer run. Time to exhaustion was recorded as a measure of exercise capacity.

Blood lipids: After the 6-week intervention, mice were fasted for 12 h, and blood was collected by retro-orbital puncture. Serum was separated by centrifugation at 3000 rpm for 15 min at 4 °C. Total cholesterol (TC), triglycerides (TG), high-density lipoprotein cholesterol (HDL-C), and low-density lipoprotein cholesterol (LDL-C) were measured using an automated biochemical analyzer (Hitachi 7180, Tokyo, Japan). Enzymatic assay kits were obtained from Nanjing Jiancheng Bioengineering Institute (Nanjing, China). All assays were performed in duplicate, and mean values were used for analysis.

#### 4.2.3. Echocardiography

After the 6-week intervention, mice were anesthetized with isoflurane (5% for induction and 1.5% for maintenance). They were positioned supine on a platform maintained at 37 °C, and the chest was depilated before ultrasound coupling gel was applied. A small animal ultrasound system with a 40 MHz linear probe was used to obtain left ventricular long-axis, papillary-level short-axis, and four-chamber views. Measurements included left ventricular end-diastolic volume (LVEDV), left ventricular end-systolic volume (LVESV), E and A waves, e’ velocity, and left ventricular ejection fraction (LVEF). Each parameter was recorded over three consecutive cardiac cycles, and mean values were used for analysis [41,42,43].

#### 4.2.4. Sample Collection

After echocardiography, mice were deeply anesthetized with isoflurane, and blood was collected by retro-orbital puncture into centrifuge tubes. Serum was separated at 3000 rpm for 15 min at 4 °C and stored at −80 °C until use. Heart and colon tissues were rapidly dissected. Portions were fixed in 4% paraformaldehyde for histological staining. Additional myocardial tissue and colonic mucosa were snap-frozen in liquid nitrogen and stored at −80 °C for molecular assays. Fresh fecal pellets were collected from surgically excised colons using sterile forceps. Samples were placed in sterile cryovials, snap-frozen in liquid nitrogen, and stored at −80 °C for microbiota analysis. All dissection instruments were sterilized before use, and samples were handled on a sterile biosafety cabinet surface to prevent exogenous microbial contamination.

#### 4.2.5. Histopathological Examination

Cardiac staining: Heart tissues fixed in 4% paraformaldehyde were dehydrated through graded ethanol (70%–80%–90%–95%–100%), cleared in xylene, embedded in paraffin, and sectioned at 5 μm. For H&E staining, sections were deparaffinized, rehydrated, stained with hematoxylin for 5 min, rinsed in running water, and stained with eosin for 2 min. They were then dehydrated, cleared, and mounted with neutral balsam. For Masson’s trichrome staining, deparaffinized and rehydrated sections were stained with Ponceau–acid fuchsin for 10 min. Sections were differentiated in phosphomolybdic acid for 5 min and stained with aniline blue for 5 min before dehydration, clearing, and mounting with neutral balsam. ImageJ (1.54 g) was used for quantitative analysis of five randomly selected fields at ×400 magnification. Measurements included inflammatory cell count, cardiomyocyte cross-sectional area, interstitial area fraction, and collagen volume fraction (collagen area/total field area × 100%). Picrosirius red staining under polarized light was used to quantify the type I/III collagen proportion.

Colonic staining: Fixed, dehydrated, and embedded colon tissues were sectioned at 5 μm. H&E staining was used to assess colonic villus morphology. For PAS-AB staining, deparaffinized and rehydrated sections underwent periodic acid oxidation for 5 min, Schiff’s reagent staining for 15 min, and Alcian Blue staining for 20 min. Sections were then dehydrated, cleared, and mounted with neutral balsam. Quantitative analysis was performed in ImageJ using five randomly selected fields at ×400 magnification. Measurements included villus thickness (vertical distance from base to tip), villus length (central axis length from base to tip), and mucus-positive area fraction (PAS-AB positive area/total field area × 100%).

All histological assays included biological replicates, with five randomly selected microscopic fields imaged per mouse to assess tissue morphology.

#### 4.2.6. Gut Microbiota and Metabolomics

16S rRNA gene amplicon sequencing: Fecal genomic DNA was extracted using the Tiangen DP302 kit. DNA purity (OD260/280 = 1.8–2.0) and concentration were assessed using a Nanodrop 2000. The V3–V4 region was amplified using primers 338F (5′-ACTCCTACGGGAGGCAGCAG-3′) and 806R (5′-GGACTACHVGGGTWTCTAAT-3′). Each 25 μL reaction contained 12.5 μL 2× Taq PCR MasterMix, 1 μL of each primer, 2 μL DNA template, and 8.5 μL ddH2O. Cycling conditions were 94 °C for 5 min; 30 cycles of 94 °C for 30 s, 55 °C for 30 s, and 72 °C for 30 s; and 72 °C for 10 min. Amplicons were checked by 2% agarose gel electrophoresis and sequenced on the Illumina NovaSeq 6000 platform using paired-end sequencing (PE250). A total of 1,411,618 paired-end raw reads were obtained from 18 samples. Quality control retained 1,288,630 clean reads, averaging 71,591 per sample. Non-chimeric reads ranged from 41,812 to 63,538 per sample, indicating sufficient sequencing depth. Low-quality and chimeric sequences were removed, and amplicon sequence variants (ASVs) were generated using QIIME 2 [44]. Analyses included α-diversity, β-diversity, and taxonomic composition at the phylum, genus, and family levels. PCoA used Bray–Curtis distances, and PERMANOVA with 999 permutations assessed between-group differences in community structure. LEfSe identified differentially abundant taxa using an LDA score > 4.0 and *p* < 0.05. These thresholds were applied to limit false-positive findings given the small sample size (*n* = 6 per group) [45]. Phylum-level taxonomy followed updated prokaryotic nomenclature from recent revisions [46].

Metagenomics: Fecal DNA from three randomly selected mice per group was fragmented by ultrasonication, and libraries were prepared with a 350 bp insert size. Libraries were sequenced on the NovaSeq 6000 platform at a depth of 10 G per sample. Trimmomatic (v0.39) was used to remove adapters and low-quality bases. Clean reads were assembled into contigs using MEGAHIT (v1.2.9), and open reading frames (ORFs) were predicted using Prodigal (v2.6.3). Functional annotation was performed with eggNOG-mapper (v2.1.9). DESeq2 (v1.38.3) identified differentially abundant genes (|log2FC| > 1, adjusted *p* < 0.05), with a focus on amino acid metabolic gene sets. KEGG enrichment analysis was performed using clusterProfiler (v4.8.3), with *p* < 0.05 defining significant enrichment and particular attention to amino acid metabolism.

Targeted amino acid metabolomics: Three mice were randomly selected from each group. Serum (50 μL) was mixed with 200 μL pre-chilled methanol (−20 °C), vortexed for 10 min, and centrifuged at 12,000 rpm for 15 min at 4 °C. The supernatant was passed through a 0.22 μm organic membrane filter and analyzed by UPLC-MS/MS. Separation used a Waters ACQUITY UPLC BEH C18 column (2.1 mm × 100 mm, 1.7 μm) at 40 °C and 0.3 mL/min. Mobile phases were 0.1% formic acid in water (A) and acetonitrile (B). The gradient was 0–0.5 min, 5% B; 0.5–5 min, 5–95% B; 5–7 min, 95% B; 7–7.1 min, 95–5% B; and 7.1–10 min, 5% B. Mass spectrometry used positive electrospray ionization (ESI+) and multiple reaction monitoring (MRM). Optimized settings were a capillary voltage of 3.0 kV, desolvation temperature of 400 °C, desolvation gas flow of 800 L/h, and cone gas flow of 50 L/h. External standard calibration curves were used to quantify plasma amino acid concentrations.

#### 4.2.7. Western Blot Analysis

Approximately 50 mg of frozen myocardial tissue or colonic mucosa was homogenized on ice in 500 μL RIPA buffer containing PMSF (1 mmol/L). After complete lysis, homogenates were centrifuged at 12,000 rpm for 15 min at 4 °C, and supernatants were collected. Protein concentrations were measured using a BCA assay kit (Beyotime, Shanghai, China) and adjusted to equal levels. Samples were mixed with 5× loading buffer at a 4:1 volume ratio, heated at 100 °C for 5 min, and stored at −20 °C.

Equal amounts of protein (30–50 μg/well) were separated by 10% SDS-PAGE at 80 V through the stacking gel and 120 V through the resolving gel. Proteins were wet-transferred to PVDF membranes (Millipore, Burlington, MA, USA) at 300 mA for 90 min. Membranes were blocked with 5% non-fat milk in TBST for 1.5 h at room temperature. After removal of the blocking solution, membranes were incubated overnight at 4 °C with primary antibodies. These were rabbit anti-mouse IL-18 (1:1000, polyclonal, Abcam, Cambridge, MA, USA), rabbit anti-mouse galectin-4 (1:500, polyclonal, Proteintech, Rosemont, IL, USA), and mouse anti-β-actin (1:5000, monoclonal, Proteintech, Rosemont, IL, USA). β-actin served as the loading control. Membranes were washed three times in TBST for 10 min each. They were then incubated for 1 h at room temperature with HRP-conjugated goat anti-rabbit or goat anti-mouse IgG (1:5000, Beyotime, Shanghai, China). After three further TBST washes, bands were visualized in a dark room using an ECL kit (Beyotime, Shanghai, China). Images were acquired with a ChemiDoc XRS+ system (Bio-Rad, Hercules, CA, USA). Band intensities were quantified using ImageJ, and target protein expression was normalized to β-actin. Each experiment was independently repeated three times. Each lane contained lysate from an individual mouse (biological replicate); the same biological sample was not reloaded as a technical replicate.

#### 4.2.8. Statistical Analysis

Statistical analyses were performed using R 4.5.1. Longitudinal data (e.g., body weight and weekly fasting blood glucose) were analyzed using two-way repeated-measures ANOVA. Group was the between-subject factor, and time was the within-subject factor. Sphericity was assessed using Mauchly’s test; when this assumption was violated, the Greenhouse–Geisser correction was used to adjust the degrees of freedom. The group effect, time effect, and group × time interaction were reported. When a significant interaction or main effect was detected, post hoc comparisons were performed using Tukey’s HSD test for between-group comparisons at each time point. For cross-sectional data (e.g., echocardiographic parameters, histological indices, endpoint blood pressure, exhaustive running time, α-diversity indices, and amino acid concentrations), one-way ANOVA followed by Tukey’s multiple comparison test was used for comparisons among multiple groups. Categorical data were analyzed using the χ^2^ test. Correlations were assessed using Pearson’s correlation coefficient. Non-normally distributed microbiota and metabolomics data were analyzed using the Kruskal–Wallis test followed by Dunn’s multiple comparison test. All data are expressed as $\bar{x}\;\pm \;SEM$. *p* < 0.05 was considered statistically significant, and *p* < 0.01 was considered highly statistically significant.

## 5. Conclusions

This study examined co-enrichment of gut *Erysipelotrichaceae* and *Desulfovibrio* and its association with metabolic alterations in HFpEF. In mice receiving HFD and L-NAME, co-enrichment of these taxa was associated with early alterations in the gut–heart axis. *Erysipelotrichaceae* enrichment coincided with greater abundance of microbial BCAA biosynthesis pathways and elevated circulating amino acids, particularly isoleucine. *Desulfovibrio* was associated with intestinal barrier integrity, altered sulfur-containing amino acid pathways, and elevated homocysteine. Gut microbiota dysbiosis also coincided with elevated circulating tryptophan and altered microbial gene modules, suggesting an additional metabolic association that warrants investigation. Together, these pathways may form a correlative network linking gut-derived signals with myocardial hypertrophy, fibrosis, and diastolic dysfunction. TXG treatment was accompanied by lower abundance of both taxa, improved intestinal barrier structure, and changes in amino acid pathways at the microbial gene and metabolite levels. These changes coincided with improved left ventricular diastolic function without impaired systolic function. The associations among bacterial taxa, amino acid metabolism, and myocardial changes provide preliminary evidence for further investigation of *G. acuta* in metabolic heart failure.

## Figures and Tables

**Figure 1:**
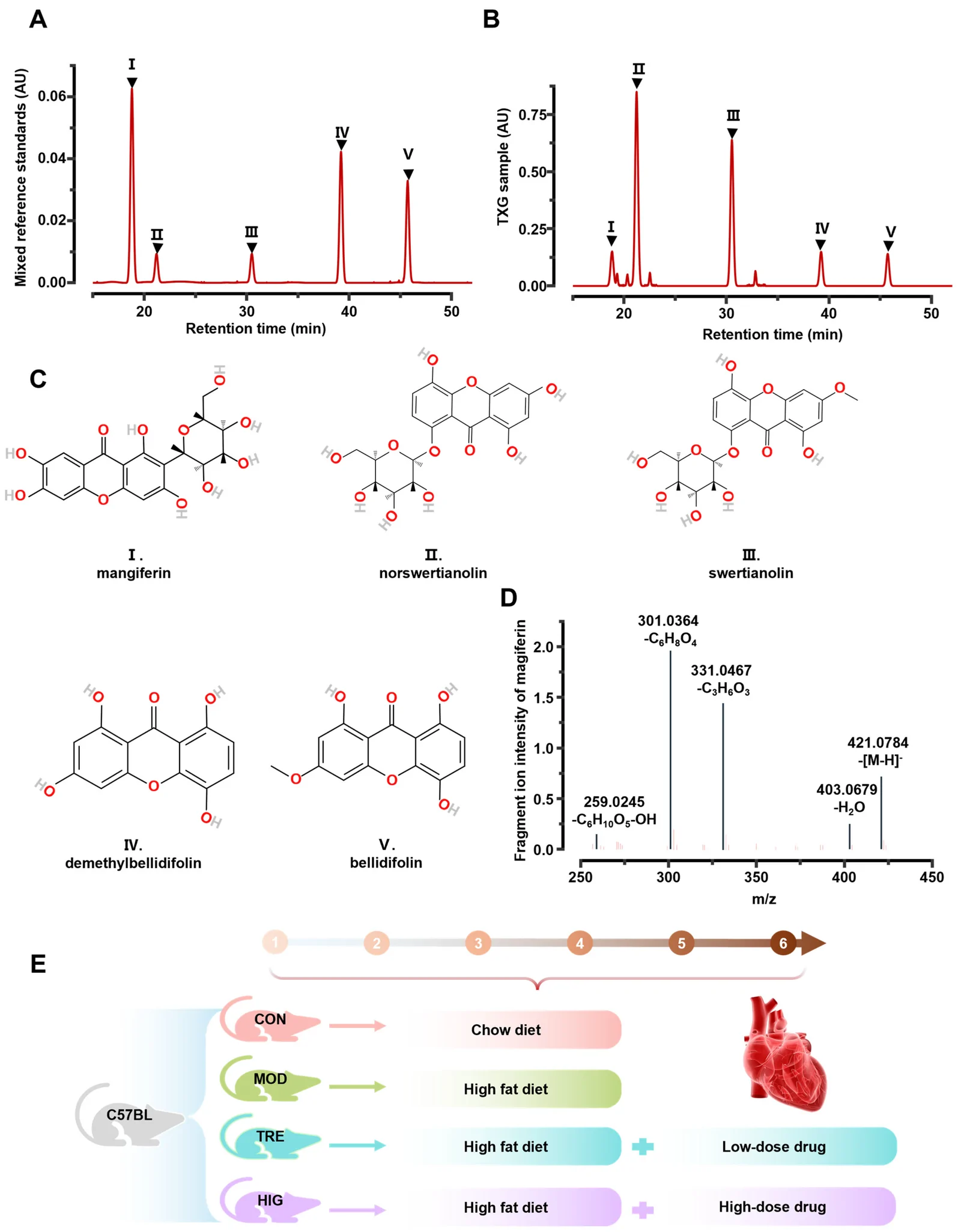
Chemical characterization of TXG and experimental design. (**A**) HPLC fingerprint of TXG showing characteristic peaks within a defined retention time range. (**B**) HPLC chromatogram of a mixed standard containing mangiferin, norswertianolin, swertianolin, demethylbellidifolin, and bellidifolin, showing correspondence with the TXG fingerprint. (**C**) Kekulé structures of the five xanthones, showing a shared dibenzo-γ-pyrone scaffold and differences in substituent type, number, and position. (**D**) Representative MS/MS spectrum of mangiferin showing characteristic neutral losses and aglycone fragment ions that support its structural identification. (**E**) Experimental design. Mice were assigned to four groups: CON (control, normal chow + water), MOD (HFD + 0.5 g/L L-NAME in water), TRE (HFD + L-NAME + TXG 4 mg/kg), and HIG (HFD + L-NAME + TXG 40 mg/kg). Echocardiographic, histological, gut microbiota, and metabolomic assessments were performed after 6 weeks of intervention.

**Figure 2:**
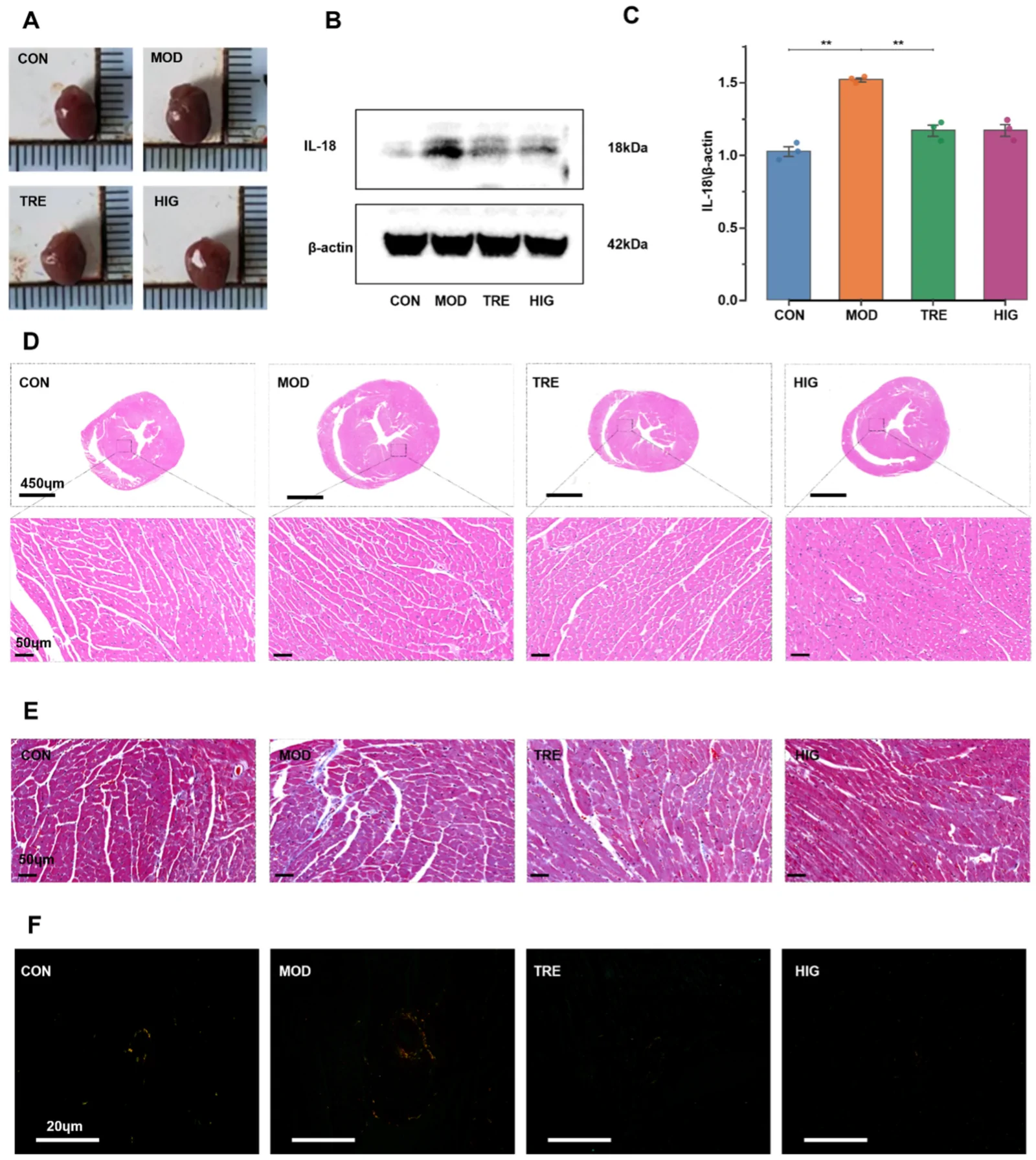
TXG attenuates myocardial injury in the HFpEF model. (**A**) Gross appearance of the heart, showing reduced cardiac hypertrophy in TXG-treated groups compared with the MOD group. (**B**) Representative Western blots of IL-18 in myocardial tissue. (**C**) Quantification of IL-18 protein levels normalized to the loading control, showing a significant increase in the MOD group that was suppressed by TXG. (**D**) Representative H&E-stained sections of myocardial tissue. The MOD group shows disrupted myocardial architecture, cardiomyocyte hypertrophy, and inflammatory cell infiltration, all of which are ameliorated by TXG. (**E**) Representative Masson’s trichrome-stained sections and quantification of perivascular collagen volume fraction (CVF). TXG significantly reduces the extensive perivascular fibrosis observed in the MOD group. (**F**) Picrosirius red staining (bright-field and polarized light) with quantification of perivascular collagen area and type I/III collagen ratio. TXG reduces perivascular collagen deposition and the type I/III ratio. Data are presented as $\bar{x}\;\pm \;SEM$. ** *p* < 0.01.

**Figure 3:**
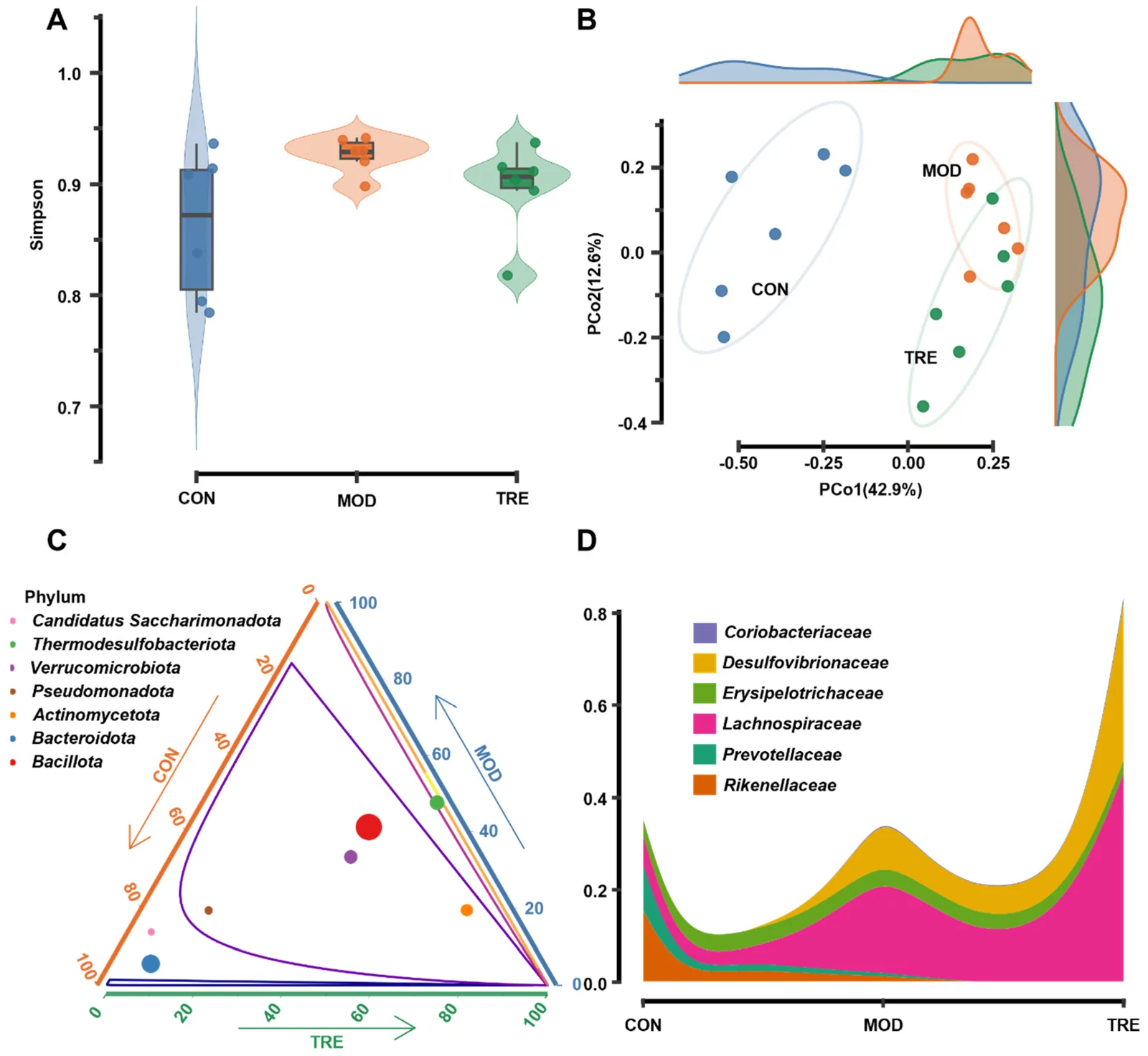
TXG is associated with altered gut microbiota structure and reduced abundance of key disease-associated bacteria in the HFpEF model. To characterize gut microbiota dysbiosis in HFpEF and changes associated with TXG, fecal samples were subjected to 16S rRNA sequencing. (**A**) Alpha diversity indices showed no statistically significant differences among the CON, MOD, and TRE groups. (**B**) Beta diversity analysis by principal coordinate analysis (PCoA) showed separation of microbial communities between the CON and MOD groups, whereas the TRE group samples shifted toward the CON group, consistent with a TXG-associated shift in community structure. Global PERMANOVA ($R^{2}=0.4814$, p=0.001) indicated significant group differences, while PERMDISP (P=0.603) provided no evidence of differences in dispersion. (**C**) Ternary plot displaying the relative abundance distribution of *Bacillota*, *Bacteroidota*, and *Pseudomonadota* among the three groups. The MOD group exhibited an increased proportion of *Pseudomonadota* and a decreased proportion of *Bacteroidota*. (**D**) River plot showing the microbiota community composition across experimental groups at the study endpoint.

**Figure 4:**
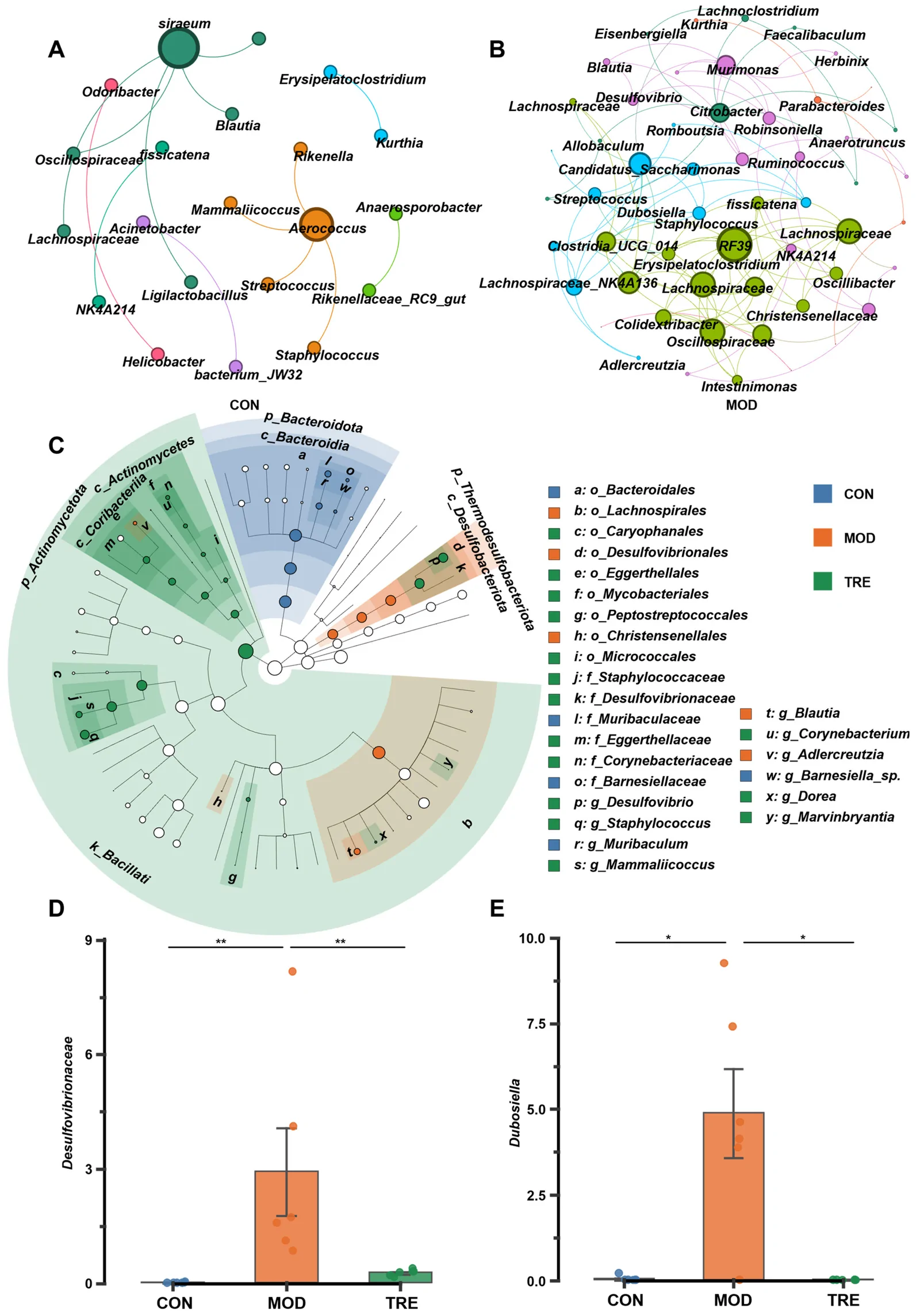
Gut microbiota correlation networks and key disease-associated taxa in the HFpEF model. (**A**,**B**) Genus-level correlation network analysis in the CON (**A**) and MOD (**B**) groups, showing increased network complexity and connection density and disruption of the balance between positive and negative correlations in the MOD group. (**C**) LEfSe cladogram displaying the phylogenetic distribution of differential taxa across taxonomic levels (LDA > 4.0). (**D**) Relative abundance of *Desulfovibrio*, a significantly enriched Gram-negative sulfate-reducing genus in the MOD group, and its altered abundance in TXG-treated mice. (**E**) Relative abundance of *Erysipelotrichaceae* (particularly the genus Dubosiella), a prominently enriched Gram-positive bacterial group in the MOD group, and its reduced abundance in TXG-treated mice. Data are presented as $\bar{x}\;\pm \;SEM$. * *p* < 0.05, ** *p* < 0.01.

**Figure 5:**
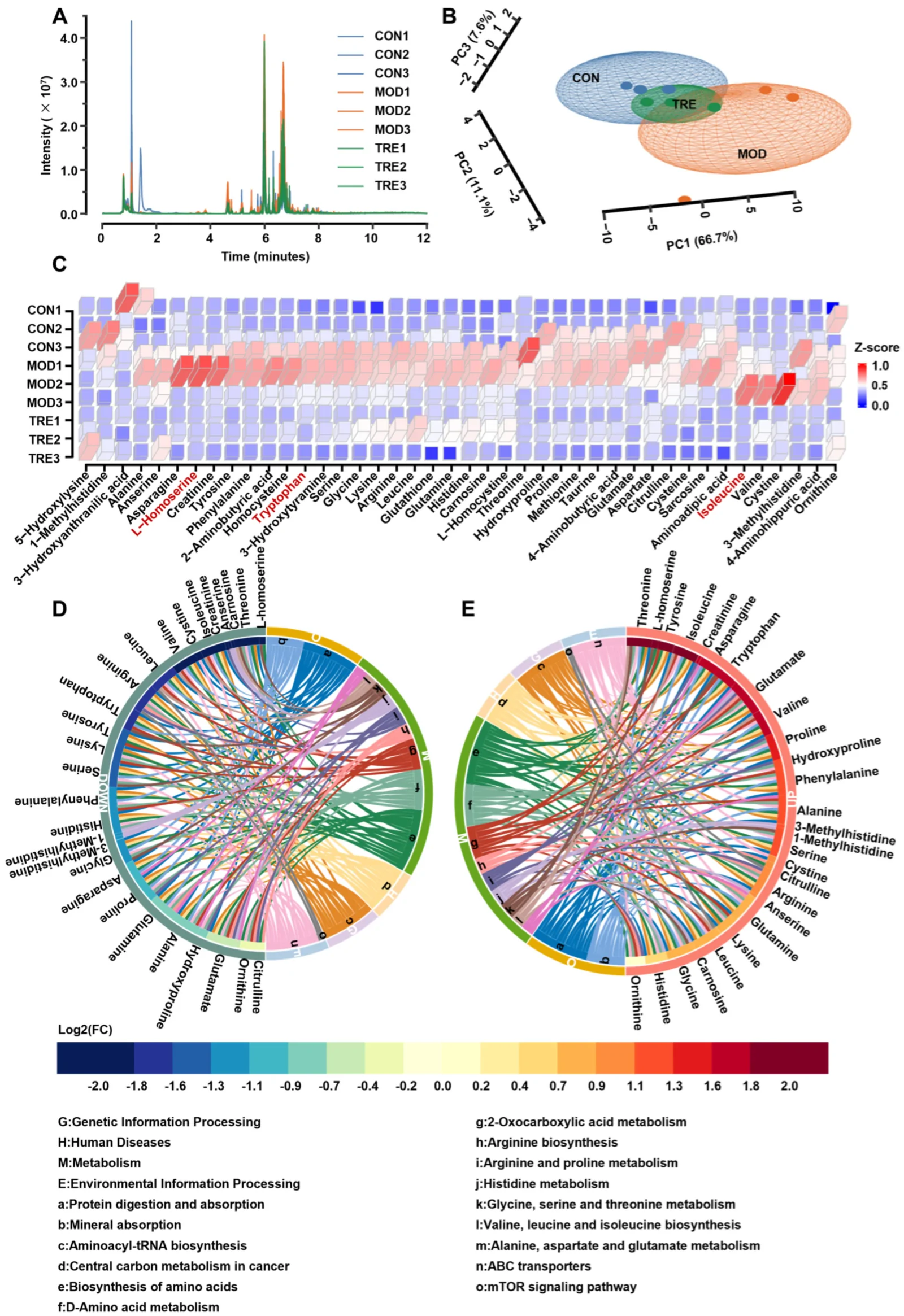

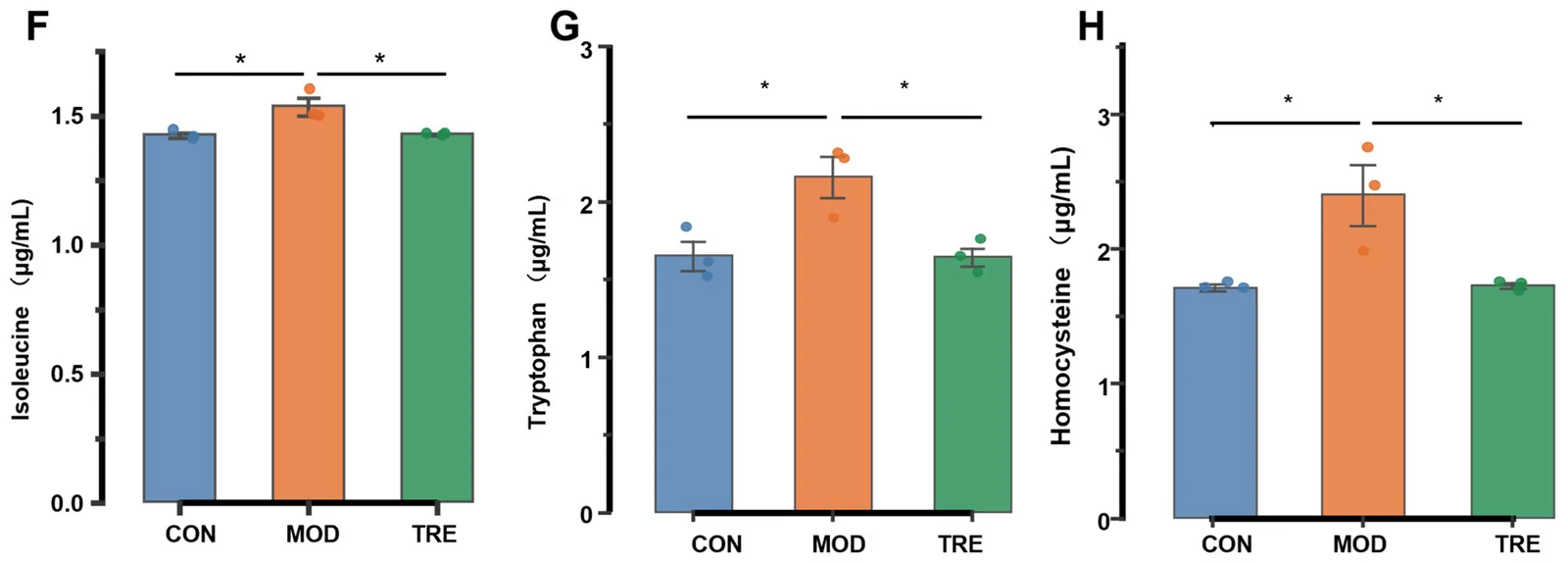
TXG is associated with altered serum amino acid profiles in the HFpEF model. (**A**) Representative total ion chromatograms (TICs) of serum samples, showing well-separated peaks, stable signals, and good retention time reproducibility. (**B**) PCA score plot demonstrating a trend toward separation among the CON, MOD, and TRE groups in serum amino acid profiles, with the TRE group profile shifting toward the CON group. (**C**) Heatmap of differentially abundant serum amino acids with hierarchical clustering, showing distinct separation of the MOD group from CON and intermediate positioning of the TRE group. (**D**) KEGG chord diagram comparing the CON and MOD groups, highlighting the pathways associated with elevated serum isoleucine levels in the MOD group. (**E**) KEGG chord diagram comparing the MOD and TRE groups, showing changes in key amino acid metabolic pathways in TXG-treated mice, including lower homocysteine levels. (**F**) Serum isoleucine concentrations (μg/mL). (**G**) Serum tryptophan concentrations (μg/mL). (**H**) Serum homocysteine concentrations (μg/mL). Individual data points from each mouse are overlaid on bar plots. Data are presented as $\bar{x}\;\pm \;SEM$. * *p* < 0.05.

**Figure 6:**
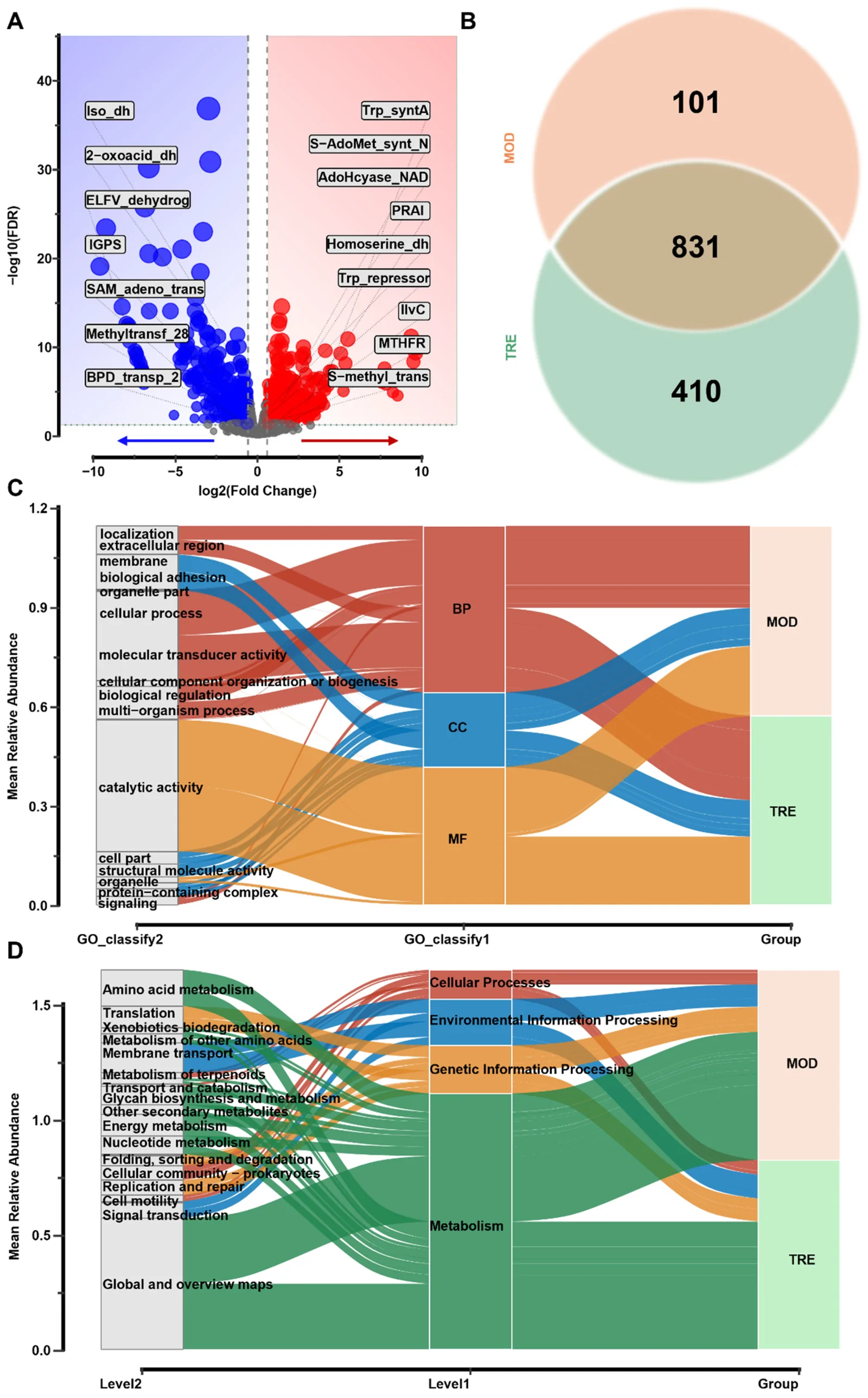
TXG is associated with altered abundance of gut microbial amino acid metabolic modules in the HFpEF model. (**A**) Volcano plot showing differentially abundant microbial genes between the MOD and TRE groups (|log2FC| > 1, FDR < 0.05), with amino acid metabolism-related genes highlighted. (**B**) Venn diagram displaying the overlap of differentially abundant genes between the MOD and TRE groups, highlighting amino acid biosynthesis and catabolism. (**C**) GO enrichment analysis illustrating the significantly enriched biological process terms in the MOD group, with the most prominent enrichment observed in amino acid metabolism-related pathways. (**D**) KEGG pathway enrichment analysis showing significant enrichment of amino acid metabolic pathways.

**Figure 7:**
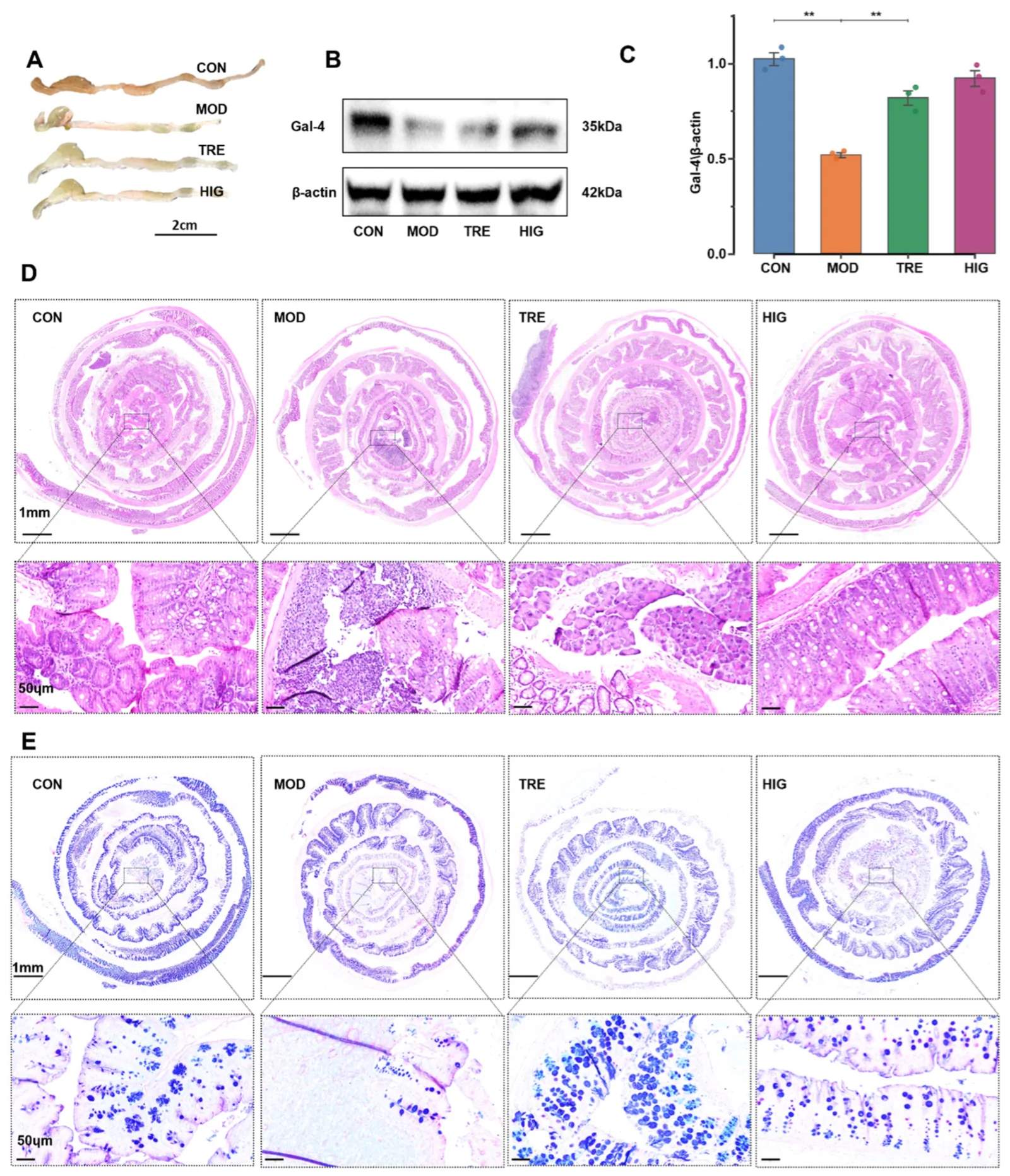
TXG is associated with reduced intestinal barrier injury in the HFpEF model. (**A**) Gross appearance of the colon. (**B**) Western blot analysis of galectin-4 (Gal-4) protein expression. (**C**) Quantification of Gal-4 protein expression levels. (**D**) Representative H&E-stained sections of colonic tissue and the corresponding inflammation score. The MOD group showed disrupted mucosal structure, inflammatory cell infiltration, and mucosal thinning, which were less severe in TXG-treated groups. (**E**) Representative PAS-AB-stained sections of colonic tissue and the quantification of the PAS-AB positive area percentage. The MOD group exhibited fewer goblet cells and reduced mucus secretion, with higher goblet cell numbers and mucus secretion in TXG-treated groups. Data are presented as $\bar{x}\pm SEM$. ** *p* < 0.01.

## Data Availability

The original contributions presented in this study are included in the article/Supplementary Material. Further inquiries can be directed to the corresponding authors.

## Supplementary Materials

The following supporting information can be downloaded at: https://www.mdpi.com/article/10.3390/ijms27198445/s1.

## Abbreviations

Abbreviation	Full Form
ASV	Amplicon Sequence Variant
BCAA	Branched-Chain Amino Acid
CON	Control Group
CVF	Collagen Volume Fraction
DBP	Diastolic Blood Pressure
EF	Ejection Fraction
FBG	Fasting Blood Glucose
FS	Fractional Shortening
Gal-4	Galectin-4
H&E	Hematoxylin and Eosin
HDL-C	High-Density Lipoprotein Cholesterol
HFD	High-Fat Diet
HFpEF	Heart Failure with Preserved Ejection Fraction
HFrEF	Heart Failure with Reduced Ejection Fraction
HIG	High-Dose TXG Group (40 mg/kg)
HPLC	High-Performance Liquid Chromatography
IL-18	Interleukin-18
KEGG	Kyoto Encyclopedia of Genes and Genomes
L-NAME	Nω-Nitro-L-arginine Methyl Ester
LDL-C	Low-Density Lipoprotein Cholesterol
LEfSe	Linear Discriminant Analysis Effect Size
LPS	Lipopolysaccharide
LVEDD	Left Ventricular End-Diastolic Diameter
LVEDV	Left Ventricular End-Diastolic Volume
LVEF	Left Ventricular Ejection Fraction
LVESD	Left Ventricular End-Systolic Diameter
LVESV	Left Ventricular End-Systolic Volume
MOD	Model Group
MRM	Multiple Reaction Monitoring
PAS-AB	Periodic Acid-Schiff and Alcian Blue
PCA	Principal Component Analysis
PCoA	Principal Coordinate Analysis
PERMANOVA	Permutational multivariate analysis of variance
SBP	Systolic Blood Pressure
SEM	Standard Error of the Mean
TC	Total Cholesterol
TG	Triglycerides
TICs	Total Ion Current
TRE	TXG Treatment Group (4 mg/kg)
TXG	Total Xanthones from *Gentianella acuta*
UPLC-MS/MS	Ultra-Performance Liquid Chromatography–Tandem Mass Spectrometry
