# Supplementary material for: Co-Enrichment of Desulfovibrio and Erysipelotrichaceae and Dysregulation of Multiple Amino Acid Pathways in HFpEF: Insights into TXG-Associated Metabolic Changes
Source: Int J Mol Sci. 2026 Sep 22;27(19):8445. doi: 10.3390/ijms27198445 (PMC13664766; doi:10.3390/ijms27198445)
Supplement: Supplementary file 1 [file ijms-27-08445-s001.zip › ijms-4503928-supplementary.pdf]

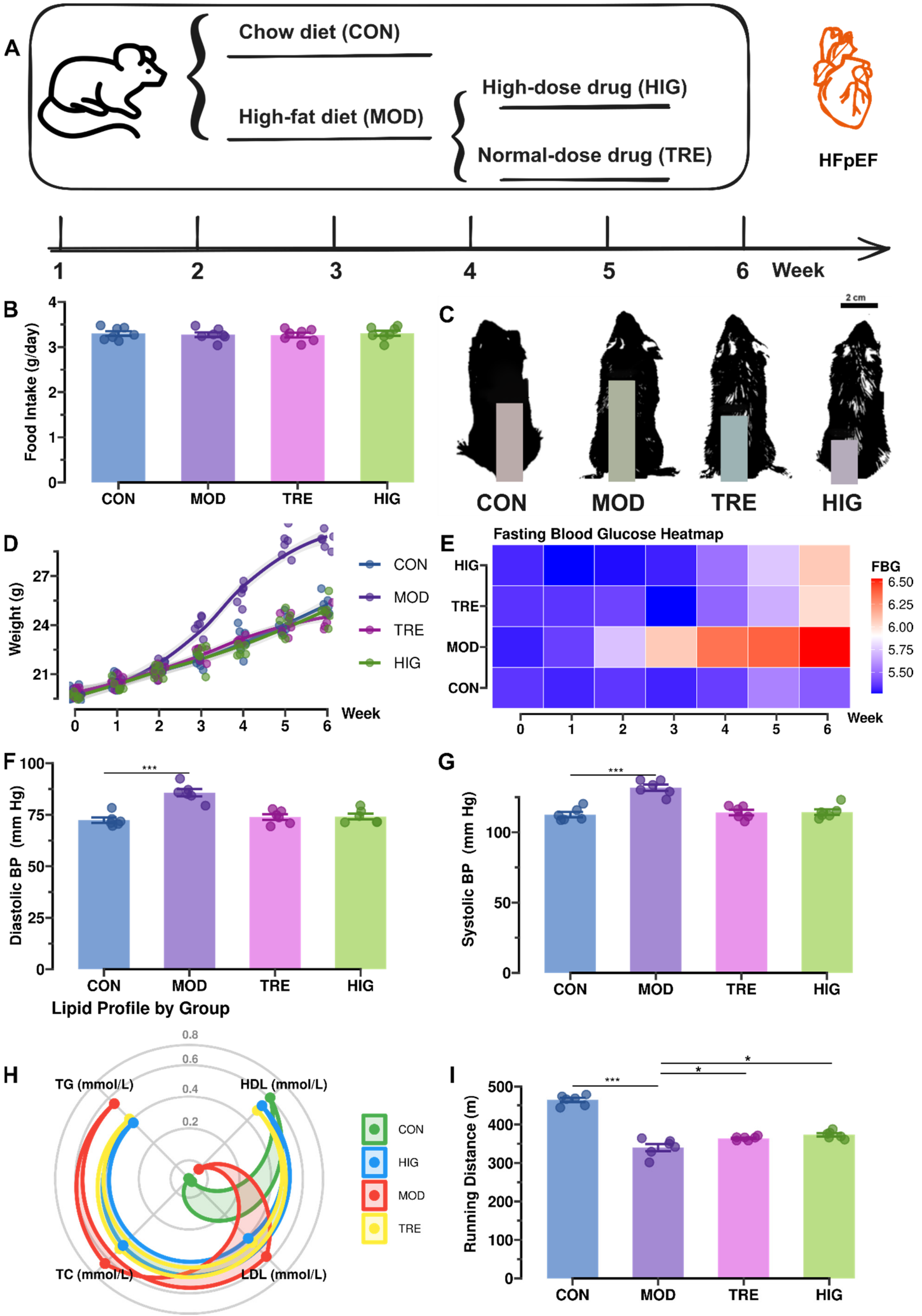

A
Chow diet (CON)
High-fat diet (MOD)
High-dose drug (HIG)
Normal-dose drug (TRE)
HFpEF
1 2 3 4 5 6 Week
B
Food Intake (g/day)
CON MOD TRE HIG
C
2 cm
CON MOD TRE HIG
D
Weight (g)
Week
CON
MOD
TRE
HIG
E
Fasting Blood Glucose Heatmap
HIG
TRE
MOD
CON
Week
FBG
6.50
6.25
6.00
5.75
5.50
F
Diastolic BP (mm Hg)
***
CON MOD TRE HIG
G
Systolic BP (mm Hg)
***
CON MOD TRE HIG
Lipid Profile by Group
H
TG (mmol/L)
HDL (mmol/L)
TC (mmol/L)
LDL (mmol/L)
CON
HIG
MOD
TRE
I
Running Distance (m)
***
*
*
CON MOD TRE HIG

Figure S1. TXG is associated with improved physiological measures in the HFpEF mouse model. (A) Experimental flowchart; (B) Average daily food intake; (C) Body weight over time; (D) Representative images of body size and corresponding quantification; (E) Heatmap of fasting blood glucose (FBG) changes; (F) Diastolic blood pressure (DBP) at endpoint; (G) Systolic blood pressure (SBP) at endpoint; (H) Radar chart of serum lipid parameters; (I) Exhaustive exercise time. Data analyzed by two-way repeated measures ANOVA (body weight, FBG) or one-way ANOVA (others) with Tukey's post hoc test, n = 6 per group. *$P < 0.05$, **$P < 0.01$, ***$P < 0.001$.

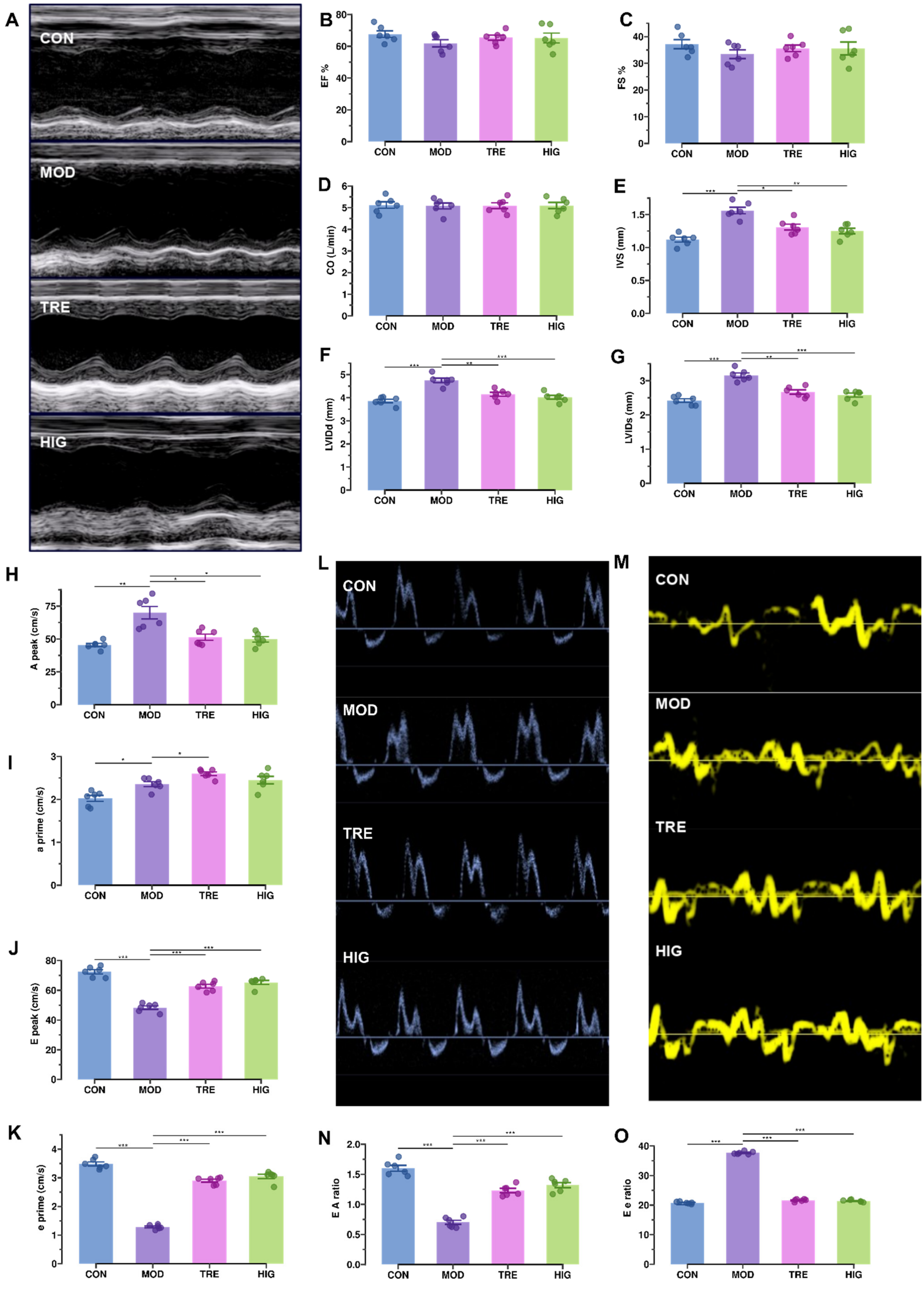
A
CON
MOD
TRE
HIG
B
EF %
C
FS %
D
CO (L/min)
E
IVS (mm)
F
LVIDd (mm)
G
LVIDs (mm)
H
A peak (cm/s)
I
a prime (cm/s)
J
E peak (cm/s)
K
e prime (cm/s)
L
M
N
E A ratio
O
E e ratio
CON
MOD
TRE
HIG

Figure. S2. TXG alleviates cardiac diastolic dysfunction in HFpEF mice. (A) Representative M-mode echocardiographic images; (B) Left ventricular ejection fraction (EF, %); (C) Fractional shortening (FS, %); (D) Cardiac output (CO, L/min); (E) Interventricular septal thickness (IVS, mm); (F) Left ventricular internal diameter at end diastole (LVIDd, mm); (G) Left ventricular internal diameter at end systole (LVIDs, mm); (H) Peak A wave velocity (A peak, cm/s); (I) Peak a' velocity (a' peak, cm/s); (J) Peak E wave velocity (E peak, cm/s); (K) Peak e' velocity (e' peak, cm/s); (L) Representative pulsed-wave Doppler images of mitral inflow; (M) Representative tissue Doppler images (lateral mitral annulus); (N) E/A ratio; (O) E/e' ratio. Data are expressed as $\bar{x} \pm SEM$. Statistical analysis was performed by one-way ANOVA followed by Tukey's post-hoc test, n=6 per group. *$P < 0.05$, **$P < 0.01$, ***$P < 0.001$.

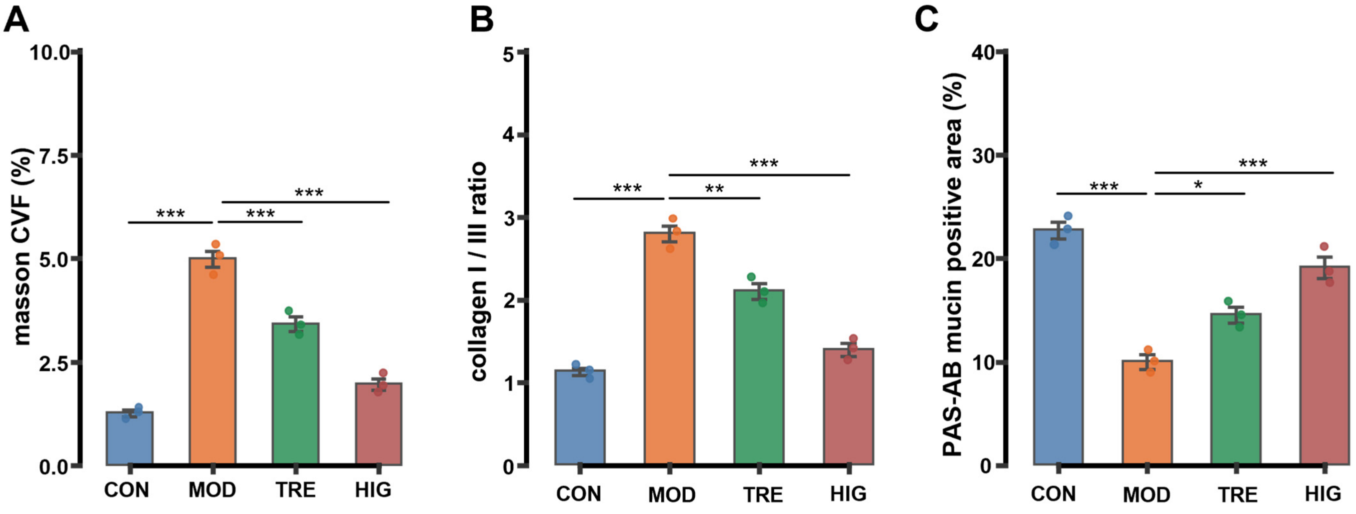

Figure S3. Quantitative histopathological assessment of heart and colon tissues. (A) Collagen volume fraction quantified from Masson's trichrome-stained heart sections. (B) Ratio of type I to type III collagen determined by picrosirius red staining under polarized light. (C) Proportion of PAS-AB-positive mucus area in colon sections. For each mouse, quantitative values were averaged from five randomly selected microscopic fields at ×400 magnification before statistical analysis. One-way ANOVA followed by Tukey's post hoc multiple comparisons test was performed. n = 3 independent mice per group. Data are expressed as $\bar{x} \pm SEM$. *$P < 0.05$, ***$P < 0.001$.
